# Coordination Models for Cancer Care in Low- and Middle-Income Countries: A Scoping Review

**DOI:** 10.3390/ijerph19137906

**Published:** 2022-06-28

**Authors:** Buhle Lubuzo, Khumbulani W. Hlongwana, Mbuzeleni Hlongwa, Themba G. Ginindza

**Affiliations:** 1 Discipline of Public Health Medicine, School of Nursing and Public Health, University of KwaZulu-Natal, Durban 4001, South Africa; hlongwanak@ukzn.ac.za (K.W.H.); hlongwa.mbu@gmail.com (M.H.); ginindza@ukzn.ac.za (T.G.G.); 2 Cancer & Infectious Diseases Epidemiology Research Unit (CIDERU), College of Health Sciences, University of KwaZulu-Natal, Durban 4041, South Africa; 3 Burden of Disease Research Unit, South African Medical Research Council, Cape Town 7505, South Africa

**Keywords:** care coordination, cancer care management, fragmented care, LMICs, review

## Abstract

Background: The coordination of cancer care among multiple providers is vital to improve care quality and ensure desirable health outcomes across the cancer continuum, yet evidence is scarce of this being optimally achieved in low- and middle-income countries (LMICs). Objective: Through this scoping review, our objective was to understand the scope of cancer care coordination interventions and services employed in LMICs, in order to synthesise the existing evidence and identify key models and their elements used to manage and/or improve cancer care coordination in these settings. Methods: A detailed search strategy was conducted, aligned with the framework of Arksey and O’Malley. Articles were examined for evidence of coordination interventions used in cancer care in LMICs. We followed the Preferred Reporting Items for Systematic Reviews and Meta-Analyses (PRISMA) Extension Guidelines for Scoping Reviews, which included a checklist and explanation. The PRISMA flow diagram was utilised to report the screening of results. Data were extracted, categorised and coded to allow for a thematic analysis of the results. Results: Fourteen studies reported on coordination interventions in cancer care in LMICs. All studies reported a positive impact of cancer coordination interventions on the primary outcome measured. Most studies reported on a patient navigation model at different points along the cancer care continuum. Conclusions: An evidence-based and culturally sensitive plan of care that aims to promote coordinated and efficient multidisciplinary care for patients with suspicion or diagnosis of cancer in LMICs is feasible and might improve the quality of care and efficiency.

## 1. Study Background

Cancer is increasingly becoming one of the leading public health problems globally [1], with significantly higher morbidity and mortality in low- and middle-income counties (LMICs) [1,2,3]. For people with symptoms that are potentially indicative of cancer, the pathway to diagnosis is complex. The pathway complexities permeate through to the point of treatment, with major difficulties observed in the referral and quality of the information communicated between providers [1,4,5]. Major difficulties noted were long distances and financial costs that resulted in barriers to accessing treatment services, and a lack of feedback negatively affected the functional referral system [4,5,6,7,8,9,10,11,12]. The cumbersome referral pathway and other access-related difficulties to the oncology services result in cancer patients in LMICs often giving up or prematurely discontinuing treatment. This inadvertently results in unreasonably lower survival rates and compromises quality of life when compared to patients from higher-income countries (HICs) [1]. The inequitable distribution of access to healthcare services between and within LMICs is a universally acknowledged challenge [13,14,15].

Multiple service providers from a number of professions, settings and social services are frequently involved in the clinical care of patients with complex and chronic diseases such as cancer [16]. As a result, there is a lack of deliberate organisation, cooperation and multi-directional sharing of information across patients, caregivers and healthcare providers, which results in fragmented and poorly coordinated care [4,5,11,17,18]. This can risk the efficiency of healthcare delivery. Therefore, in this study, we make a case for coordinated care among multiple providers to avoid inefficient duplication of diagnostic testing, delayed transfer transmission and confusion about unclear care plans [19]. Health policy documents have emphasised the need to develop organisational models to improve the integration of cancer coordination around patient needs [20,21,22], and this study builds on existing policy directives to propose an appropriate cancer care model.

Care coordination strategies are of great interest as they have the potential to improve the quality of healthcare, efficiency and optimal patient health outcomes. Given the high cost and complexity of patients’ growing needs from diagnosis to survivorship, cancer patients require care that is integrated across providers and settings over time [16,23,24]. Care coordination is a multidimensional concept and a critical aspect of healthcare that spans the continuum of care by ensuring quality care for better patient outcomes [25]. In HICs, coordination strategies have been successfully implemented to facilitate the patient’s journey along the cancer care pathway [13,26,27]. It has become a global priority area for improving patient healthcare from prevention through to disease management [28]. While approaches to coordinating care may vary widely, the general intent of these strategies is to facilitate the delivery of the right healthcare services in the right order, at the right time and in the right setting [12,17,25,27,29,30,31,32,33,34,35,36,37,38,39,40,41,42,43,44,45]. 

In the absence of consensus around what constitutes an integrated care plan, especially for cancer patients in LMICs, we conducted this scoping review to understand the scope of cancer care coordination interventions and the services employed in these settings to help patients overcome barriers, navigate care pathways and receive timely and appropriate care. An initial search was performed to determine whether a previous review addressing this topic has been completed or is in progress. The PubMed database was searched, and no complete or in-process reviews focussing on mapping coordination models for cancer care in LMICs were found. With this scoping review, we aim to synthesise literature on cancer care and its coordination. 

## 2. Methodology

### 2.1. Study Design

We retrieved published peer-reviewed articles and grey literature (unpublished scholarly studies such as theses and/or dissertations) on cancer care coordination models in LMICs. This scoping review was conducted as part of a multi-phase study aimed at proposing a model for cancer care coordination interventions that can be used to guide and achieve coordinated access to lung cancer care in selected public healthcare facilities in KwaZulu-Natal (KZN), South Africa. Scoping reviews are a recent method of reviewing evidence-based research outputs, particularly in health and other disciplines [46]. They help to understand research fields that are mostly in early stages by allowing the mapping of key concepts, sources and types of available evidence that leads to identifying research gaps within the existing literature [47]. 

A scoping review framework developed by Arksey and O’Malley guided this review [47]. The framework provides review guidance for the following five stages: (I) identify the research question; (II) identify relevant studies; (III) select eligible studies; (IV) chart the data; and (V) collate, summarise and report the results. The presentation of the results follows the Preferred Reporting Items for Systematic Reviews and Meta-Analyses (PRISMA) Extension Guidelines for Scoping Reviews (PRISMA-ScR), which include a checklist and explanation [48]. 

### 2.2. Identification of the Research Question

To determine the research question’s eligibility for a scoping review project, we applied the PCC (Population, Concept, and Context) framework recommended by the Joanna Briggs Institute 2015 [49,50]. The framework is illustrated in Table 1. The proposed scoping review seeks to address the following research question:

*What are the coordination models and their key elements used for cancer care in LMICs?*

### 2.3. Information Sources and Search Strategy for the Identification of Relevant Studies

#### 2.3.1. Databases 

The databases (and platforms) chosen for this review were PubMed, American Doctoral Dissertations via EBSCO host, Union Catalogue of Theses and Dissertations (UCTD) and SA e-Publications via SABINET Online, World Cat Dissertations and Theses via OCLC and other supplementary information sources, such as Google Scholar and e-hand-searching [51]. We conducted a comprehensive literature search through a keyword search for relevant articles from these databases, and all study designs were included. We utilised evidence published by primary studies and grey literature, which have shown significant results for all study designs. Reviews were not eligible for inclusion; however, reference lists of relevant reviews and full-text articles were screened for more relevant primary studies. 

#### 2.3.2. Search Strategy

For this review, the search strategy was comprehensive and covered areas of cancer care coordination in LMICs with assistance from a librarian. The eligibility criteria were designed to focus the study only on the articles that address issues described in the research question. The keywords consisted of free text and Medical Subject Headings (MeSH) terms. Key search terms used for building the search strategy included “cancer coordination”, “cancer care”, “cancer management”, “developing countries”, “under-developed countries”, “low- and middle-income countries”, “sub-Saharan Africa”, “care plans” and “integrated care”. The literature search was not restricted by year of publication. Boolean terms (AND/OR) were used to separate our keywords. Language restrictions were not applied to minimise the risk of excluding relevant studies. 

#### 2.3.3. Search Management

All retrieved articles from the electronic databases and e-hand searches, deemed to meet the inclusion criteria, were then exported to EndNote (version 20, Stanford, CT, USA), a reference management software, which was used to create a virtual library [52]. Deduplication followed immediately after transferring all the retrieved article records to EndNote.

### 2.4. Selection of Eligible Studies

The eligibility criteria were developed according to the relevant elements of the PCCd-T (study design-Time) framework guidance for undertaking a scoping review to ensure that the proposed research question’s boundaries are clearly defined. Eligible studies were included after two reviewers had independently and reproducibly evaluated them; studies had to present evidence on either of the factors, as illustrated in Table 2. Any disagreements between the two reviewers were resolved through discussions or by engaging a third reviewer.

### 2.5. Inclusion and Exclusion Criteria

This review includes studies that contain information about cancer care and a coordination intervention in an LMIC. The research team used the World Bank’s categorisation of countries by income [67] to determine the income status of the countries identified in the search corresponding to the year of data collection. The following principles were used to determine the studies that met the inclusion criteria:

(1) Studies presenting evidence published in an LMIC.
(2) Studies reporting evidence on cancer care.
(3) Studies presenting evidence on coordination interventions or models.

Studies with the following characteristics were excluded:

(1) Studies that were not conducted in LMICs.
(2) Studies with no evidence on cancer care coordination.
(3) Studies with no evidence on coordination models/interventions.

### 2.6. Selection of Sources of Evidence

Our study selection was conducted in three stages: title, abstract and full-article screening. 

#### 2.6.1. Title Screening

Firstly, the principal investigator (PI) conducted the electronic database (and platforms) search and screened titles of identified articles guided by the study eligibility criteria. Potential articles from the database search were exported to EndNote version 20 for further assessment [68] and duplicates were removed. 

#### 2.6.2. Abstract Screening

Following deduplication, the PI and a second reviewer independently screened abstracts of articles identified as relevant. Articles not meeting the inclusion criteria for this review were excluded. Discrepancies in the reviewers’ responses at this stage were resolved via a discussion until an agreement was reached. 

#### 2.6.3. Full-Text Screening

**Figure 1:**
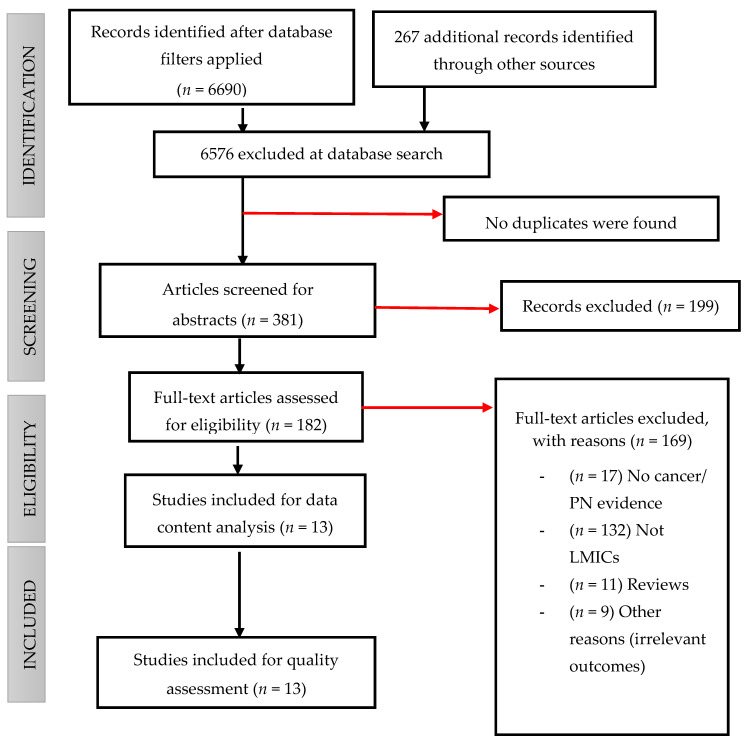
PRISMA-ScR flow chart demonstrating the screening results of each stage.

Lastly, full texts were sought for all studies appearing to meet the inclusion criteria, and a final selection was made. Disagreements were resolved by consensus and by consulting a third reviewer. Both abstract and full-article screening were guided by a screening tool that factored all aspects of the inclusion/exclusion criteria and the PCC elements. Detected differences between reviewers were resolved by a third reviewer called to adjudicate discrepancies between the two reviewers. A flow chart of the study selection procedure at each stage of the review was prepared, detailing when exclusion occurred (Figure 1).

### 2.7. Charting the Data

Data extraction was conducted independently by the PI, guided by a predefined yet flexible data extraction form designed using Google Forms. The data charting form was developed and utilised as a guide to extract the background information from each of the included studies. It aimed to ensure that all required information was captured efficiently and accurately, thereby minimising the risk of missing information. We extracted data on the following: author and year of publication, aim of the study, country of the study, cancer type and target population, coordination model and study design (Table 2). The PI synthesised the data and prepared the final manuscript. The results of the search are reported in full in this final report and presented in a PRISMA flow diagram [48] (Figure 1).

### 2.8. Collating, Summarising and Reporting the Results

The chosen analytical approach was of a narrative nature. For coding and analysing data from the selected articles, thematic analysis of the extracted data was conducted. NVivo version 12 [69] was used to identify emerging themes from the included articles, and our reporting was then structured around these themes. 

### 2.9. Quality Assessment of the Included Studies

We used the mixed method appraisal tool (MMAT) version 2018 to evaluate the quality of the included articles [53]. Using MMAT, we appraised the methodological quality of five categories of research: qualitative research, randomised controlled trials, non-randomised studies, quantitative descriptive studies and mixed methods studies [53]. The following percentage scores were used to grade the quality of evidence: (i) ≤50% represented low-quality evidence, (ii) 51–75% represented average-quality evidence and (iii) 76–100% represented high-quality evidence.

### 2.10. Ethical Considerations

No ethical approval was required for this literature-based study.

## 3. Results 

### 3.1. Screening Results 

#### 3.1.1. Title Screening

After applying database filters, the initial electronic database searches identified 6690 potentially eligible articles. An additional 267 articles that the primary search strategies could not capture were retrieved from other sources (Google Scholar, American Doctoral Dissertations via EBSCO host, Union Catalogue of Theses and Dissertations (UCTD) and SA e-Publications via SABINET Online, World Cat Dissertations and Theses via OCLC). All these articles were screened for titles, and 6576 articles were not selected because they did not meet our inclusion criteria. 

#### 3.1.2. Abstract Screening

Following deduplication, the PI and a second reviewer independently screened the abstracts of articles identified as relevant. A total of 381 articles were eligible for abstract screening. Articles not meeting the inclusion criteria for this review were excluded (*n* = 199).

#### 3.1.3. Full-Text Screening

The team of screeners further screened 182 full-text articles and excluded 169, mainly because they were not in LMICs, they were reviews or evaluated other diseases. In the end, a total of 13 articles met our inclusion criteria and were taken forward to data content analysis.

**Figure 2:**
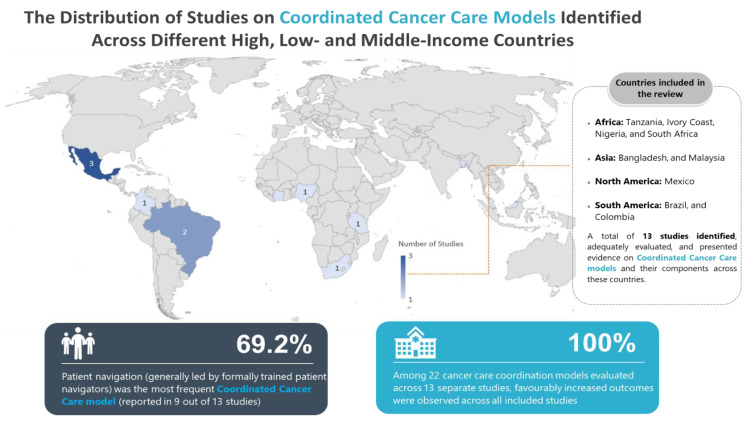
The world map presenting countries included in the review. Countries are listed according to the region and in alphabetical order (figure generated by the first author using PowerPoint).

Figure 1 displays the PRISMA flow diagram, which demonstrates the review of 182 texts, resulting in 13 studies being included in the synthesis. They presented evidence on 22 cancer care coordination models and their components across ten countries (see Figure 2).

### 3.2. Characteristics of the Included Studies

The characteristics of the 13 studies, including the profile of study participants, country, geographic setting, type of cancer targeted by the intervention, platform of care, coordinated care model under study and healthcare provider involved, are summarised in Table 2. Table 2 describes the characteristics of all the included studies in greater detail, and Figure 2 below provides an overview of the countries where the 13 studies were conducted. 

#### 3.2.1. Study Designs and Settings of the Included Articles

The most commonly used study design (*n* = 3) was a cohort analysis study conducted in private and public healthcare facilities and health-related NGOs [58,65,66]. The other common design (*n* = 3) used in this sample were randomised controlled trials (RCT; [55,57,64]), followed by cross-sectional surveys (*n* = 2 [54,59]), pilot studies (*n* = 2 [62,63]), mixed method studies (*n* = 1 [61]) and exploratory studies (*n* = 1 [56]) (Table 2). Three out of fourteen studies were conducted in community-based healthcare [56,57,62] and working with health-related NGOs [65], and one study using a smartphone-empowered community health workers (CHWs) navigators’ model of care [57]. However, the majority (9 out of 13 studies) were conducted in urban areas [54,55,56,58,60,62,63,64,65], and only three were conducted in rural areas [56,57,61] (see Table 2).

#### 3.2.2. Quality of Evidence from Included Studies

The study team assessed each article included in the review for rigor, sample size and study design. No articles were excluded from the review based on these characteristics. All of the included studies that underwent methodological quality assessment achieved a high-quality score between 76% and 100%. The overall evidence was considered to have minimal risk of bias. A summary of the critically assessed domains is provided as supplementary material (Supplementary S1). The overall quality of evidence (or certainty in the findings) for each outcome collected was assessed based on study methodological quality (see Table 2).

### 3.3. Key Themes

#### 3.3.1. Cancer Care Coordination Models: Types

All studies identified themselves as quality management models that were designed to improve management of cancer patients in LMICs [54,55,56,57,58,59,60,61,62,63,64,65,66]. The majority of articles included in this study (11 out of 13 studies) reported on patient navigation models [54,55,56,57,58,59,60,63,64,65,66], with one intervention focussing on training navigators [61] and the other being a coordinated program of screening and early diagnosis intervention [62]. The most common health workers to carry out services were professionally trained nurses (*n* = 8 [55,58,59,60,61,62,65,66]; 61.5%). Some of the services outlined in these studies included facilitating and fast-tracking linkages to follow-up services [58,59,62,63,65]; health promotion [57]; assuring adherence to treatment [54,55,57,58]; and addressing health disparities and alleviate institutional, socioeconomic and personal barriers to timely cancer care [56,58,62,63,65,66].

These interventions were most commonly offered in individual sessions (*n* = 11 [54,55,56,57,58,59,60,63,64,65,66]; 84.6%) or by use of mobile health platforms (a smartphone-empowered navigator model; *n* = 1 [57]). Seven studies in this scoping review focused mainly on women’s cancers, predominantly breast cancer (*n* = 7 [56,57,58,59,62,65,66]; 53.8%), followed by cervical cancer (*n* = 2 [54,62]; 15.4%), highlighting another gap in the evidence on cancer care coordination. Ten studies (76.9%) implemented coordination intervention in hospital-based healthcare [54,55,58,59,60,61,63,64,65,66]. Many of the healthcare facilities were public (seven studies, 53.8%), two were private and others were not specified. 

#### 3.3.2. Cancer Care Coordination Models: Key Elements

Twelve studies (92.3%) reported on coordinating cancer care and services [54,55,56,57,58,59,60,62,63,64,65,66]. Across the various types of coordination models, care was coordinated by multi-disciplinary teams [63,64], involving mostly trained nurses (*n* = 8 [55,58,59,60,61,62,65,66]; 61.5%), and others involving social workers [62], cancer survivors, volunteers and community health workers [57,63]. Six studies (46.2%) occurred at the screening and diagnosis level [54,57,58,62,63,65], seven studies (53.8%) occurred at the treatment level [54,55,56,58,59,60,66] and two studies (15.4%) were at the supportive care level [56,64]. There was no intervention that spanned across all stages of the cancer journey from diagnosis through to survivorship in one study.

An assessment of patient needs was observed across the various types of interventions. The development of a navigation program for cancer patients in one included study resulted in the structuring of a program model suited to the needs of patients and the operation of referral service in Brazilian oncology [60]. The barriers associated with the use of coordination models originated mostly at the patient level and included most prominently a lack of geographic access to health facilities, health system barriers, poor distribution of services, sociocultural barriers limiting access to healthcare [56,58,59,60,63,65] and affordability and availability of cancer services [54,59,63,66]. 

#### 3.3.3. Cancer Care Coordination Models: Key Outcomes

All studies reported a positive impact of cancer coordination interventions on the primary outcome measured. Broadly, the outcomes used to assess the impact of intervention can be categorised as process, implementation and clinical outcomes. Five articles (38.5%) reported on process outcomes, which included the coordination of appointments, follow-up and referrals [54,58,59,62,65], and the ability to overcome psychosocial barriers [56]. Better diagnostic timelines were shown, and a reduction was distinguished in the time elapsed from diagnosis to the initiation of treatment [54,58,59,65]. Three articles (23.1%) reported on implementation outcomes, which included acceptability and feasibility of the intervention and where implementation of the interventions could be applied to LMIC populations [54,56,62]. Six articles (46.2%) reported on clinical outcomes, which included improved screening rates, patient retention in treatments and clinical advice [54,55,57,58,64,66]. 

One article (7.7%) focused on testing the efficacy of online training intended to improve trainee confidence in carrying out core patient navigation tasks by providing preliminary data that support feasibility and utility of using this training [61]. Moreover, one study showed that patient navigation can significantly improve access to early supportive and palliative care, advanced care planning and pain control for patients with cancer [63].

## 4. Discussion

While there is a growing emphasis on the use of coordination models to guide the organisation and delivery of care for cancer patients, our understanding of the components and key facilitators of integrated care uptake is still in its early stages [70]. Coordination of care ensures that due processes are followed and relevant structures are held accountable. Communication and integration of services and settings need to be considered in any plan of care and align with the patient and family preferences and goals. [25,27,30,32,35,36,37,38,40,41,43,44,45]. 

Existing literature states that care coordination helps reduce in-hospital complications [71] and improves the quality and efficiency of care [54,56,58,59,60,63,64,65,72]. Our results demonstrate that an evidence-based and culturally sensitive coordination model for patients with suspicion or diagnosis of cancer in LMICs is feasible, and that such a model may lead to an improved time to referral for specialised cancer care [54,57,58,59,61,62,63,65]. It is important to note that health systems in LMICs differ in terms of the availability of resources and accessibility to services. Strategies such as providing specialist services closer to patients might improve access to timely care and adherence to treatment and clinical advice. Nonetheless, there is a paucity of research on how cancer patients’ coordinated care interventions are developed and executed, what activities they include, and whether organisational and system-level characteristics permit their acceptance in LMICs [70]. How services are delivered can have an impact on the effectiveness, efficiency and equity of health systems, especially in countries with low-resourced and weak health systems. Questions remain as to which types of interventions might effectively translate across resource settings and health systems contexts. 

Furthermore, improvement in cancer care outcomes was greater for cancer detection and diagnosis [54,57,58,59,62,65], rapid treatment initiation and adherence [54,56,57,58,59,60,66] and end-of-life care [63] than for cancer survivorship. Across the continuum of cancer care, patient navigation was the most recurrent care coordination intervention [54,55,56,57,58,59,60,63,64,65,66]. Minority participants from rural or underserved locations made up just over a quarter of the reviewed studies [56,57,66], indicating that replication in other underprivileged populations is possible. In the future, studies will need to consider challenges to accessing services such as lack of geographic access, a well-known gap in cancer care in LMICs, sociocultural barriers and weak health systems. Program/model characteristics such as the use of volunteer or clinical navigators were identified as contributors to patterns of model concordance [56].

This review was unable to identify a single study that spanned across all stages of the cancer journey from diagnosis through to survivorship. Similarities were, however, noted across the different types of models included in this review in terms of the design features and core components observed, indicating the potential to leverage these shared elements to create a coordination intervention that spans across stages of the cancer journey. Patient navigation was found to be effective at all stages of cancer treatment and is thought to play an essential role in lowering barriers to cancer care in LMICs [54,55,56,57,58,59,60,63,64,65,66]. In the 13 studies included in this review, the types of patient navigation services that were offered mirrored those provided in HICs [54,57,58,59,60,62,63,65,66], where the inclusion of patient navigation services is associated with improvements in access to timely diagnosis, treatment adherence [54,57,58,66] and follow-up, especially for vulnerable and marginalised populations [63], as supported by a scoping review that provided a comprehensive overview of patient navigation interventions in cancer care in LMICs [73]. 

We sought to present the current level of knowledge about cancer care coordination and the components needed to improve it in LMICs in this review. As a result, we hope that the findings of this scoping review will contribute to cancer care literature and policy guidelines in LMICs in general.

### 4.1. Strengths and Limitations

This review aimed at mapping evidence on cancer care coordination interventions that have been implemented in LMICs. In general, it is difficult to make generalisable conclusions regarding the applicability of the findings to lower-resourced countries or health systems, given marked differences in the settings amongst LMICs. However, this overview may help policymakers and other stakeholders to identify evidence-informed strategies to improve the delivery of services. Future studies targeted at planning, implementing and assessing interventions to improve the quality of care for cancer patients will benefit from the findings of this study. This overview may help populations make decisions about delivery arrangements for improving care coordination across healthcare systems in LMICs, especially for patients with multiple chronic diseases.

Despite our thorough search technique, we may not have found all studies that incorporated coordinating components in cancer care in LMICs. Although our title screening included a wide range of databases, the overall search strategy may have been biased toward public health and social sciences. Searching other bibliographic databases may have yielded additional published studies. It is possible that we could have missed a number of relevant reviews since our searches were restricted to primary studies only. While our review included any article published in any language, our search was conducted using only English terms. Despite the generally relevant keywords/terms used while searching for relevant articles in different databases, other terms may also exist as reference to cancer coordination interventions. 

Despite these limitations, we have confidence that our search strategy was comprehensive in reviewing public health and social sciences literature on cancer care coordination. We believe that the articles in this issue contribute to the global advancement of cancer health services and continue to push the boundaries of care in low-resource contexts.

### 4.2. Recommendations for Future Research

From this review, we have identified several areas that should be addressed in future research:

(1) Measure the full economic costs of care coordination intervention.
(2) Evaluate the effectiveness of the interventions identified.
(3) Develop new theoretical models and interventions to enhance patient self-management.

## 5. Conclusions

The need for a care coordination model to help patients navigate the complex cancer care system is highlighted in this study. Our findings show that coordination-focused models are a practical and novel way to overcome healthcare system constraints in LMICs, and that they can reduce referral delays to cancer centres, thereby allowing patients to receive timely care. For all identified coordination models, we identified gaps in primary research related to uncertainty about the applicability of the evidence to very low-income countries and health systems.

## Figures and Tables

**Table 1:** PCC Framework.

CRITERIA	DETERMINANTS
**Population**	Cancer patients All cancer types
**Concept**	Cancer coordination models and their elements *Coordination:* The organisation of the different elements of cancer care or services so as to enable the healthcare team and patient to work together effectively. *Coordination models:* Enabling a useful interaction/intervention used to express coordination strategies that lead to the coherent behaviour of interacting entities. *Elements:* A set of qualities, important characteristics of collaboration, coordination and communication.
**Context**	LMICs

**Table 2:** Characteristics of 13 included studies matching eligibility criteria, presenting evidence on coordinated cancer care models identified across different LMICs.

Author, Publication Year	Aim of the Study	Country, Geographic Setting	Study Design, Platform of Care	Type of Cancer, Profile of Study Participants	Coordinated Care Model, Healthcare Provider and Stage of Care	Descriptive Scoring Using MMAT Criteria [53]	Significant Findings
Koneru, 2017 [54]	To identify barriers to cervical cancer screening and treatment and determine acceptance toward peer navigators to reduce barriers.	Tanzania, urban	Cross-sectional study, HIV clinics in Dar es Salaam	Cervical cancer: - Women with HIV infection aged ≥19 years, diagnosed with cervical cancer	Peer navigation: - “Peer” not specified - Screening, diagnosis and treatment stage	The evidence consisted of a non-blinded, non-randomised trial. There was not a good representation of the population; however, all groups were appropriately measured, relevant confounders were accounted for and the intervention was administered as planned.	PNs were found to be highly acceptable and represented a novel approach to cervical cancer screening and treatment barriers.
Koffi, 2019 [55]	To improve clinical management of malignant lymphoma patients in LMICs.	Ivory Coast, urban	Prospective randomised study, Abidjan University Medical Center (Ivory Coast)	Malignant lymphoma: - Newly diagnosed patient with HL or NHL, or endemic Burkitt lymphoma aged 5 to 75 years	Ambulatory Medical Assistance (AMA), a PN-based procedure: - Nurse navigator - Treatment stage	Participants were randomly assigned to an experimental or control group. This reduces the potential for bias and the impact of variables outside the researcher’s control. The intervention was implemented well and participants adhered to the assigned intervention. Collected data addressed the research question.	AMA was found to be a simple and relatively inexpensive procedure that could be applied to LMIC patients and had the potential to efficiently reduce refusal or abandonment of therapy and improve observance in treated patients.
Gunn, 2014 [56]	To determine how closely a published navigation model reflects navigation practice in breast cancer patient navigation programs.	Multiple sites, urban and rural	An exploratory study, hospital-based and community-based healthcare	Breast cancer: - 10 programs from a set of 40 funded by a single foundation	Navigation model (8 urban hospital-based models and two rural community-based models) - Led by volunteer clinician and healthcare team - Treatment and supportive care stage	The study design used answered the research question. Study findings were derived from the data and were appropriately reported. However, observational data represent a limitation on the conclusiveness of the findings.	Program characteristics such as the use of volunteer or clinical navigators were identified as contributors to patterns of model concordance.
Ginsburg, 2014 [57]	To demonstrate proof of concept for a smartphone-empowered community health worker (CHW) model of care for breast health promotion, clinical breast examination and patient navigation in rural Bangladesh.	Bangladesh, rural	Randomised controlled trial, community-based healthcare	Breast cancer: - Women aged ≥25 years	A smartphone-empowered CHW “navigators” model of care: - Community health workers - Screening and diagnosis stage	Randomisation was appropriately performed. Researchers managed trial participants’ engagement with the study, including exposure to the intervention. Although the outcome data were incomplete, reporting of reasons was provided.	The CHWs guided by smartphone applications were more efficient and effective in breast health promotion than the control group. CHW “navigators” were most effective in encouraging women with an abnormal breast examination to adhere to advice regarding clinic attendance.
Yeoh, 2018 [58]	To assess the feasibility of PN in a state hospital in Malaysia, and report the impact on diagnostic and treatment timeliness for patients in its first year of implementation.	Malaysia, urban	Cohort study, hospital-based healthcare	Breast cancer: - Patients diagnosed with breast cancer	Established patient navigation: - Led by nurse navigators professionally trained in general nursing, oncology, breast care and surgery - Screening, diagnosis and treatment stage	The target population was represented well, all groups were appropriately measured, relevant confounders were accounted for and the intervention was administered as intended.	When combined with a state-run breast clinic, PN is a feasible option for addressing barriers to cancer care, better diagnostic timeliness and lower treatment default.
Rohsig, 2019 [59]	To describe the outcomes of a pioneering nurse navigation program in a private, non-profit hospital in southern Brazil.	Brazil, urban	Cross-sectional, retrospective study, cancer centre in a private, non-profit hospital in southern Brazil	Breast cancer: - Two hundred sixty-three female patients with breast cancer were referred to the nurse navigation program	Nurse navigation: - Led by professionally trained nurse navigators - Treatment stage	The target population was represented well; however, the authors did not use measurement instruments as data were collected electronically from medical records. Relevant confounders were accounted for and the intervention was administered as planned.	The navigation program and hospital quality indicators showed a reduction in the time elapsed from diagnosis to the start of treatment from 24 days in 2014 to 18 days in 2017.
Pautasso, 2020 [60]	To develop a navigation program for cancer patients, based on the model proposed by the GW Cancer Institute at George Washington University, adapted to the reality of a Brazilian High-Complexity Oncology Centre.	Brazil, urban	Convergent care research, CACON: High-Complexity Oncology Centre	Patients with head and neck cancer - Head and neck cancer patients of all ages, most of whom were 61–75 years old (43%)	Navigation program - Led by professionally trained nurse - Treatment stage	Study design effectively addressed the research questions; benefits of both methods were integrated. However, an evaluation of the developed program would have provided the strongest evidence.	The development of a navigation program for cancer patients resulted in the structuring of a program model suited to the needs of patients and the operation of reference service in Brazilian oncology.
Chidebe, 2021 [61]	To test the efficacy of an online navigation training designed to improve trainee confidence in performing core patient navigation tasks among Nigerian nurses, patient advocates and cancer survivors.	Nigeria, rural	Mixed method, the National Hospital Abuja	Targeted all types of cancers - Nurses, advocates and cancer survivors	Online navigation training: *effectiveness* - Nurses, patient advocates and cancer survivors - N/A	Different components of the study were appropriately integrated and adhered to quality criteria of the methods. Outputs of the integration of qualitative and quantitative components are adequately interpreted in this pilot study.	This study provided preliminary data that support the feasibility and utility of using the GW Cancer Center online patient navigation training in non-U.S. settings.
Sardi, 2019 [62]	To implement an efficient healthcare model that can be replicated in other underserved populations.	Colombia, urban	Pilot study, through community healthcare	Breast and cervical cancers - Women	A coordinated program of screening and early diagnosis - Nurses, medical assistants, psychologists and social workers - Screening and diagnosis	This pilot study documents multifaceted comprehensive data from personal experiences, meetings and discussions. Although details on the methodology are not presented well, we considered this fair as it is a documentation of a pilot study.	To date, more than 1500 women have benefited from this initiative, which has expanded to other regions.
Chavarri-Guerra, 2019 [63]	To evaluate a patient navigation program to reduce referral time to cancer centres for underserved patients with suspicion or diagnosis of cancer at a general public hospital in Mexico City.	Mexico, urban	A pilot study, general second-level public hospital in Mexico City	Targeted all types of cancers - Seventy patients (median age 54, range 19–85) who were underserved and uninsured participated	Patient navigation: *feasibility* - A trained patient navigator - Screening and diagnosis stage	The target population was represented well; there was no control group, but the intervention group was appropriately measured; relevant confounders were accounted for throughout and the intervention was administered as intended.	This study shows that PN represents a feasible and innovative solution to overcome healthcare system barriers in LMICs by reducing referral times to cancer centres for patients with a suspicion of cancer or with cancer.
Soto-Perez-de-Celis, 2021 [64]	To study whether patient navigation increased access to multidisciplinary supportive care among Mexican patients with advanced cancer.	Mexico, urban	A randomised controlled trial, public hospital	Metastatic tumours - Patients aged ≥18 years with metastatic tumours ≤6 weeks from diagnosis	Patient navigation - Led by a multidisciplinary team of HCWs - Supportive care stage	Randomised controlled trials are excellent at answering questions about the effects of an intervention on a population. Randomisation was performed appropriately, but blinding of participants and researchers was not possible. This increases the impact of biases on the outcome of the trial. The participants adhered to the assigned intervention and outcome data were complete.	The study shows that patient navigation can significantly improve access to early supportive and palliative care, advanced care planning and pain control for patients with cancer.
Tamez-Salazar, 2020 [65]	To shorten the system delays that can be influenced through patient navigation	Mexico, urban	Cohort study, private and public healthcare facilities and health-related NGOs	All breast cancer patients who contacted Alerta Rosa from December 2017 to December 2019 were included in this study	Novel Alert and Navigation Breast Cancer Program - BC-dedicated NGO members, a registered nurse and volunteer radiology technicians - Screening and diagnosis stage	Intervention was administered well. Confounders were adequately accounted for in the analysis. Target population was not represented well, but reasons were stipulated as a lack of accessibility of the program and inclusion of other centres.	Alerta Rosa is a navigation program in Nuevo Leon that successfully reduces the health system interval from initial contact to breast cancer diagnosis.
Čačala, 2021 [66]	To determine if breast cancer research workers de facto impacted patients’ adherence to treatment by comparing groups with and without these patient navigators	South Africa, rural	Retrospective cohort study, public hospital oncology centre and tertiary surgical unit	Breast cancer - Breast cancer patients offered chemotherapy as their initial treatment, excluding those who had surgery as a primary treatment	Patient navigation - Led by breast cancer research workers (BCRWs) who had no formal training as patient navigators (professional nurse and a social worker worked closely with the surgeon and oncologist, acquiring “real-time” training) - Treatment stage	Participants were representative of the target population, and measurements were appropriate. The intervention was administered as intended, but there were incomplete outcome data. As a result, the detailed analysis of the results was compromised.	In this study, BCRWs as de facto BCNs were beneficial for BC patient care, improving chemotherapy compliance and therapeutic surgical interventions. This highlighted the need for BCNs in the management of BC patients in South Africa.

## Data Availability

All data generated or analysed during this study are included in the published review article.

## Supplementary Materials

The following supporting information can be downloaded at: https://www.mdpi.com/article/10.3390/ijerph19137906/s1, File S1: Part I: Mixed Methods Appraisal Tool (MMAT), version 2018.

## Abbreviations

CIDERU	Cancer & Infectious Diseases Epidemiology Research Unit
HICs	High-income countries
LMICs	Low- and middle-income counties
MeSH	Medical Subject Headings
MLCCP	Multinational Lung Cancer Control Programme
PCC	Population, Concept, and Context
PI	Principal investigator
PRISMA	Preferred Reporting Items for Systematic Reviews and Meta-Analyses
PRISMA-ScR	PRISMA Extension for Scoping Reviews
RCT	Randomised controlled trials
UCTD	Union Catalogue of Theses and Dissertations
UKZN	University of KwaZulu-Natal
WHO	World Health Organisation
